# TFEB and TFE3 regulate STING1-dependent immune responses by controlling type I interferon signaling

**DOI:** 10.1080/15548627.2025.2487036

**Published:** 2025-04-20

**Authors:** Pablo J Tapia, José A Martina, Pablo S Contreras, Akriti Prashar, Eutteum Jeong, Dominic De Nardo, Rosa Puertollano

**Affiliations:** a Cell and Developmental Biology Center, National Heart, Lung, and Blood Institute, National Institutes of Health, Bethesda, MD, USA; b Department of Biochemistry and Molecular Biology, Monash Biomedicine Discovery Institute, Monash University, Clayton, Victoria, Australia

**Keywords:** Autophagy, immune response, lysosomes, STING1, TFE3, TFEB

## Abstract

STING1 is an essential component of the innate immune defense against a wide variety of pathogens. Whereas induction of type I interferon (IFN) responses is one of the best-defined functions of STING1, our transcriptomic analysis revealed IFN-independent activities of STING1 in macrophages, including transcriptional upregulation of numerous lysosomal and autophagic genes. This upregulation was mediated by the STING1-induced activation of the transcription factors TFEB and TFE3, and led to increased autophagy, lysosomal biogenesis, and lysosomal acidification. TFEB and TFE3 also modulated IFN-dependent STING1 signaling by controlling IRF3 activation. IFN production and cell death were increased in TFEB- and TFE3-depleted iBMDMs. Conversely, TFEB overexpression led to reduced IRF3 activation and an almost complete inhibition of IFN synthesis and secretion, resulting in decreased CASP3 activation and increased cell survival. Our study reveals a key role of TFEB and TFE3 as regulators of STING1-mediated innate antiviral immunity.

**Abbreviation:** ACOD1/IRG1, aconitate decarboxylase 1; cGAMP, cyclic guanosine monophosphate-adenosine monophosphate; CGAS, cyclic GMP-AMP synthase; DMXAA, 5,6-dimethylxanthenone-4-acetic acid; EIF4EBP1, eukaryotic translation initiation factor 4E binding protein 1; GABARAP, GABA type A receptor-associated protein; HSV-1, herpes simplex virus type; iBMDMs, immortalized bone marrow-derived macrophages; IFN, type I interferon; IFNB, interferon beta; IKBKE, inhibitor of nuclear factor kappa B kinase subunit epsilon; IRF3, interferon regulatory factor 3; LAMP1, lysosomal associated membrane protein 1; LAMP2, lysosomal associated membrane protein 2; MTORC1, mechanistic target of rapamycin kinase complex 1; RPS6, ribosomal protein S6; STING1, stimulator of interferon response cGAMP interactor 1; TBK1, TANK binding kinase 1; TFE3, transcription factor binding to IGHM enhancer 3; TFEB, transcription factor EB.

## Introduction

The CGAS-STING1 pathway constitutes one of the first lines of defense against pathogen infection [1]. Host and pathogen-derived DNA are recognized by CGAS (cyclic GMP-AMP synthase), which activates to synthesize cyclic guanosine monophosphate-adenosine monophosphate (cGAMP) [2,3]. Binding of cGAMP, as well as bacterial cyclic dinucleotides, to STING1 (stimulator of interferon response cGAMP interactor 1) causes conformational changes and oligomerization, resulting in its transport from the endoplasmic reticulum (ER) to the Golgi apparatus and endosomes [4,5]. STING1-mediated recruitment of TBK1 and IRF3 to Golgi membranes induces IRF3 phosphorylation, dimerization, and transport to the nucleus to promote type I interferon (IFN) production [6]. CGAS-STING1 signaling also induces expression of inflammatory cytokines and chemokines via NFKB/NF-κB activation [7]. A recent report suggested that STING1 may function as a channel, promoting proton leakage from the Golgi that results in non-canonical LC3 lipidation and inflammasome activation [8].

STING1 can also induce autophagy to further promote efficient clearance of microbes and cytosolic DNA [9,10]. It was reported that the increased formation of ER-Golgi intermediate compartment (ERGIC) vesicles following trafficking of active STING1 out of the ER, together with CGAS-mediated LC3 lipidation of ERGIC membranes and post-Golgi vesicles, promotes autophagosome formation. Accordingly, mouse *CGAS/cgas-*knockdown macrophages show not only reduced production of IFNs, but also diminished autophagy activation [11]. Furthermore, STING1 triggered autophagy is essential for the antiviral response against herpes simplex virus type 1 (HSV-1) [12,13] and Zika virus/ZYKV [14], as well as for clearance of pathogenic bacteria, such as *Mycobacterium tuberculosis*, *Listeria innocua*, and *Staphylococcus aureus* [15,16]. Interestingly, inhibition of TBK1, IRF3 or IKBKE/IKKε does not prevent STING1-induced autophagy [9]. The fact that some ancient organisms, such as sea anemones, activate autophagy responses via a STING1 homolog that lacks the TBK1 activation domain [9] further suggests that this pathway may have been an essential component of the CGAS-STING1 antimicrobial response even before the evolutive appearance of interferons. Finally, it is important to consider that autophagy and lysosomes are also essential for the attenuation of the STING1 signaling, as they participate in the degradation of cytosolic DNA, as well as several signaling components, including CGAS, IRF3, TBK1 and STING1 itself [17–20]. This termination is crucial to prevent excessive immune response and consequent tissue damage.

Lysosomal and autophagic genes are transcriptionally upregulated in response to stress. The transcription factors TFEB and TFE3 translocate from the cytosol to the nucleus in response to a wide variety of stress conditions, including nutrient deprivation, organelle damage, oxidative stress, and pathogen infection. Active TFEB and TFE3 increase expression of multiple lysosomal, autophagic, metabolic, and stress response genes to restore cellular homeostasis. The important role of TFEB and TFE3 in innate immune responses has become evident in recent years. TFEB-induced autophagy is critical to limit growth of *Mycobacterium tuberculosis* [21] and *Salmonella typhimurium* [22], and TFEB and TFE3 activation has been observed following human immunodeficiency virus/HIV and beta-coronavirus infection [23,24]. The contribution of TFEB and TFE3 to cell defense is not limited to the modulation of the autophagic/lysosomal pathway, as these transcription factors also participate in production of inflammatory cytokines [25–28], dendritic cell migration [29], and monocyte and macrophage differentiation [30–32].

In this study we show that TFEB and TFE3 participate in STING1 signaling. STING1 activation prevented interaction of TFEB and TFE3 with RRAG GTPases, inducing their translocation from the cytosol to the nucleus. This resulted in increased number and activity of lysosomes, as well as autophagy induction. TFEB and TFE3 function was also critical to modulate levels of active IRF3, a process that had important consequences in IFN production and cell viability.

## Results

### STING1 activation results in upregulation of lysosomal and autophagic genes

**Figure 1.**
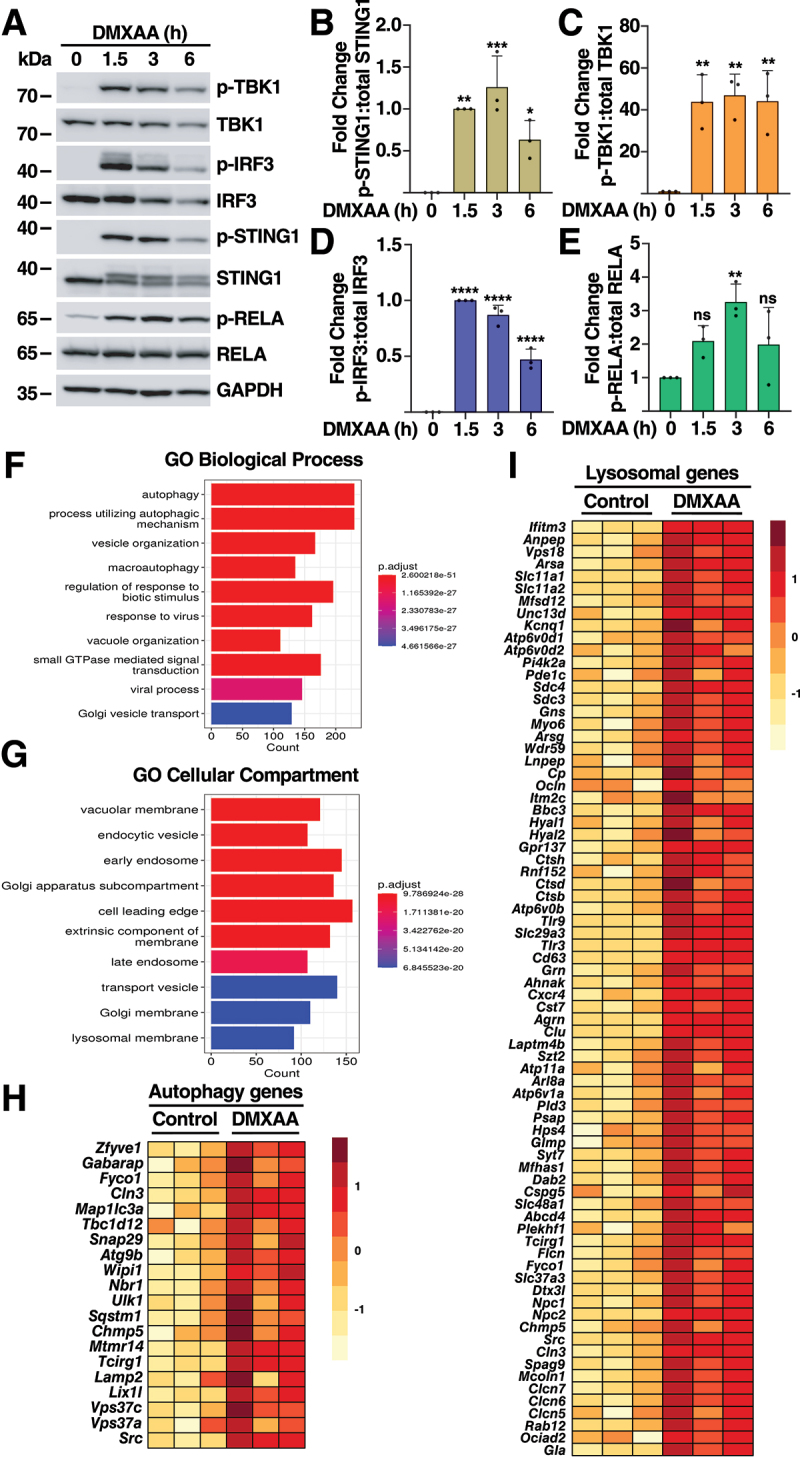
Activation of the STING1 pathway enhances expression of autophagy and lysosomal genes. (A) Representative immunoblot showing activation of the STING1 pathway in iBMDM cells treated with DMXAA 10 µg/ml for 1.5, 3, and 6 h. Immunoblots are representative of three independent experiments. (B-E) Quantification of protein levels showing p-STING1:total STING1 ratio expressed as fold increase relative to 1.5 h (B), p-TBK1:total TBK1 ratio as fold increase (C), p-IRF3:total IRF3 ratio expressed as fold increase relative to 1.5 h (D), and p-RELA/p65:total RELA/p65 ratio as fold increase (E). Data are presented as mean ± SD. (ns) not significant, (*) *p* < 0.05, (***) *p* < 0.001, and (****) *p* < 0.0001 (one-way ANOVA followed by Dunnett’s multiple comparison posttest). (F, G) Gene ontology analysis of differentially expressed genes (DEGs) from RNA-Seq data of iBMDM cells treated with DMXAA 10 µg/ml for 3 h, categorized by biological process (F) and cellular compartment terms (G). (H, I) Heatmaps showing differentially expressed genes from RNA-Seq analysis of iBMDM cells treated with DMXAA 10 µg/ml for 3 h, highlighting autophagy (H) and lysosomal gene expression (I).

To further identify cellular pathways regulated by STING1, we analyzed changes in the transcriptomic landscape of immortalized murine bone marrow-derived macrophages (iBMDMs) in response to treatment with the STING1 agonist DMXAA. As expected, the agonist induced rapid phosphorylation of STING1, TBK1 and IRF3 (Figure 1A–D), as well as activation of the NFKB/NF-κB pathway (Figure 1A,E). As a result, we observed a robust increase in the expression and secretion of IFNB/IFNβ (Figure S1A and S1B). We then performed RNA-seq analysis of iBMDMs treated with DMXAA for three hours. The comparative principal component analysis showed that activation of STING1 by DMXAA induced profound transcriptional changes when compared with control cells (Figure S1C). In total we found 10,599 differentially expressed genes, 5,408 upregulated and 5,191 downregulated (Table S1). Gene ontology (GO) analysis of upregulated genes using Biological Process and KEGG pathways revealed many categories related to immune response against viral and bacterial infection (Figures 1F and S1D); and immune genes were the most robustly upregulated in response to STING1 activation (Table S1). This upregulation was confirmed by quantitative RT-PCR (qPCR). As seen in Figure S1E-I, the expression of several inflammatory cytokines and chemokines was dramatically increased by DMXAA. Interestingly, our GO analysis revealed that among the most relevant categories were also many related to Autophagy and Vacuole Organization (Figures 1F and S1D). Similar results were obtained when we performed GO analysis of Cellular Compartment, with most categories linked to the endo/lysosomal pathway (Figure 1G). Heat-maps of selected genes further illustrated the broad upregulation in the expression of many autophagic and lysosomal components (Figure 1H,I). Interestingly, over 270 of genes upregulated by STING1 activation were previously identified as TFEB targets (Table S2). Overall, our data identify an unexpected new role of STING1 signaling in the transcriptional regulation of lysosomal biogenesis and autophagic processes.

### TFEB and TFE3 translocate to the nucleus in response to STING1 activation

**Figure 2.**
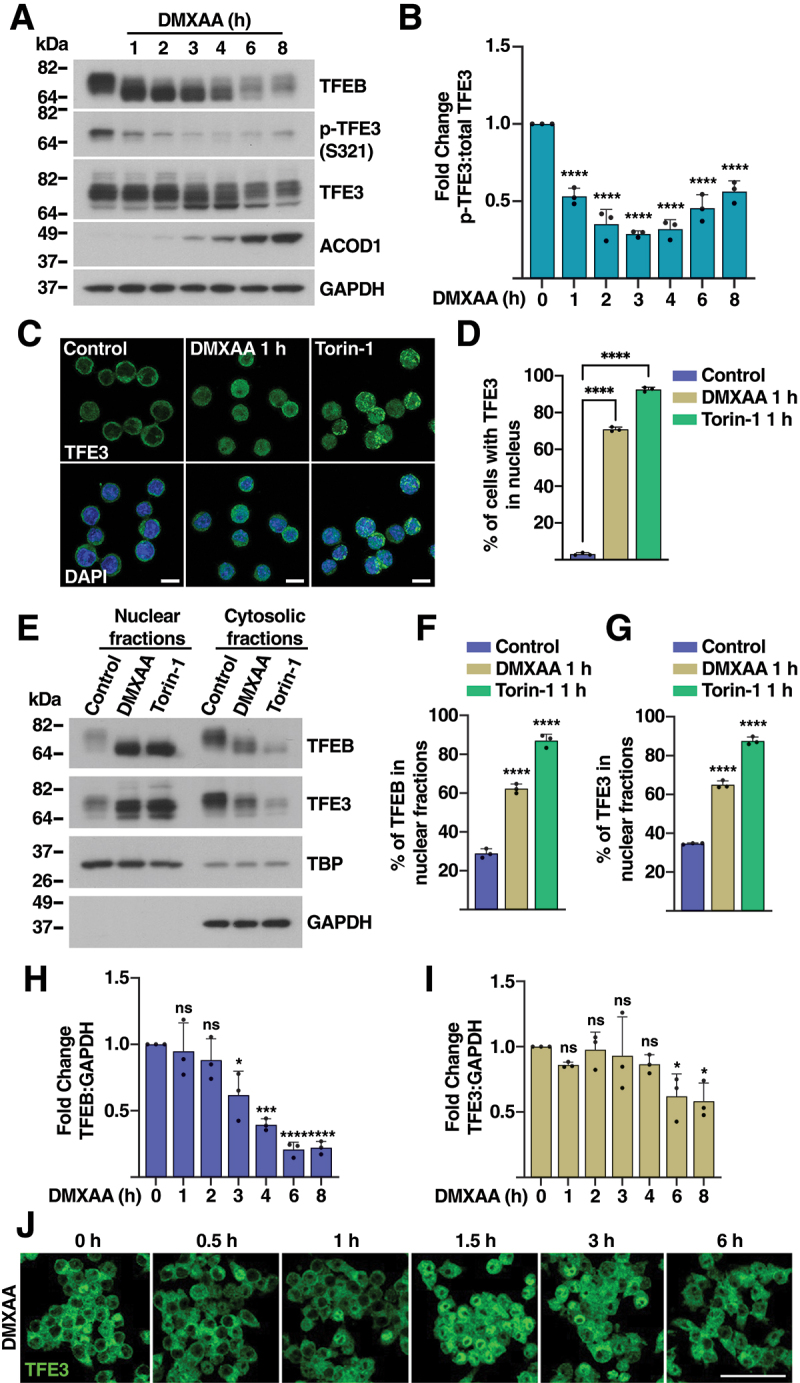
**TFEB and TFE3** are activated in response to STING1 stimulation. (A) Immunoblots showing expression levels of TFEB, TFE3, p-TFE3 (S321), and ACOD1/IRG1 in iBMDM cells treated with DMXAA (10 µg/ml) for the indicated times. Immunoblots are representative of three independent experiments. (B) Quantification of p-TFE3 (S321):total TFE3 ratio based on immunoblots shown in (A) (*n* = 3). (C) Immunofluorescence confocal microscopy of iBMDMs showing the subcellular distribution of TFE3 (green) and nuclei (blue, DAPI) in response to treatments with 10 µg/ml DMXAA or 250 nM torin-1 for 1 h. Scale bars: 10 μm (*n* = 3). (D) Quantification of the number of cells with TFE3 in the nucleus in response to treatment with 10 µg/ml DMXAA or 250 nM torin-1 for 1 h as shown in (C). In all conditions more than 300 cells per experiment were analyzed. (E) Representative immunoblot analysis of protein lysates from iBMDM cells treated with 10 µg/ml DMXAA or 250 nM torin-1 for 1 h that were subjected to subcellular fractionation (*n* = 3). (F, G) Quantification of nuclear protein levels from immunoblots in (E), showing the percentage of TFEB (F) and TFE3 (G) (*n* = 3). (H, I) Quantification from immunoblots in (A), showing total proteins levels of TFEB (H) and TFE3 (I) (*n* = 3). (J) Representative immunofluorescence images of TFE3 (green) in RAW 264.7 cells treated with DMXAA (10 µg/ml) for 0.5, 1, 1.5, 3, and 6 h. Scale bar: 50 µm (*n* = 3). Data in (B), (D) and (F-I) are presented as mean ± SD. (ns) not significant, (*) *p* < 0.05, (***) *p* < 0.001, and (****) *p* < 0.0001 (one-way ANOVA followed by Dunnett’s multiple comparison posttest).

Given the well-known role of TFEB and TFE3 in lysosomal biogenesis and autophagy regulation, we investigated whether these transcription factors may be activated by STING1. For this, we treated iBMDMs with DMXAA for different periods of time and assessed TFEB and TFE3 electrophoretic mobility and phosphorylation. It is well established that different stressors, such as nutrient deprivation or MTORC1 inactivation, cause a noticeable change in the electrophoretic mobility of TFEB, which is indicative of dephosphorylation and consequent activation [33–36]. Interestingly, treatment with DMXAA caused a rapid increase in TFEB mobility, suggesting activation of this transcription factor (Figure 2A). Likewise, phosphorylation of TFE3 at S321 was significantly reduced by DMXAA (Figure 2A,B). Phosphorylated TFE3-S321 mediates interaction with YWHA/14-3-3 and causes TFE3 retention in the cytosol. Therefore, TFE3-S321 dephosphorylation is a readout of TFE3 nuclear translocation and activation [36]. Accordingly, we observed accumulation of TFE3 in the nucleus following STING1 activation (Figure 2C,D). Torin-1, an MTORC1 catalytic inhibitor, was used as positive control (Figure 2C,D). These results were further corroborated by subcellular fractionation. As seen in Figure 2E–G, treatment with either DMXAA or torin-1, caused a significant increase in the amount of TFEB and TFE3 in nuclear fractions. Notably, prolonged DMXAA treatment resulted in TFEB and TFE3 degradation (Figure 2A,H,), suggesting that their activation is transient and highly regulated.

To further corroborate our results, we activated STING1 in RAW264.7 macrophages. As expected, incubation with DMXAA induced trafficking of STING1 from the ER to the Golgi as early as 30 min following treatment with the agonist (Figure S2A). At 3 h treatment, most STING1 had left the Golgi and was delivered to lysosomes for degradation (Figure S2B and S2C). Phosphorylation of TFE3 and TFEB was clearly reduced at 1 h DMXAA treatment but partially recovered at later times, once again suggesting temporary activation (Figure S2D and S2E). This was further confirmed by immunofluorescence analysis. As seen in Figures 2J and S2F, nuclear accumulation of TFE3 and TFEB was maximal at 1.5 h DMXAA treatment in RAW264.7 cells, while at 6 h treatment the transcription factors were located predominantly in the cytosol.

### DMXAA prevents interaction of TFEB with RRAG GTPases

Next, we investigated the mechanism of DMXAA-induced TFEB and TFE3 activation. Previous reports have suggested that upregulation of the enzyme ACOD1/IRG1 in activated macrophages leads to the generation of itaconate, which can antagonize the binding of TFEB to YWHA/14-3-3 to induce nuclear translocation [22]. However, our data showed that efficient induction of ACOD1/IRG1 occurred at late times of DMXAA treatment (Figure 2A), while TFEB and TFE3 dephosphorylation was readily observed after just 1 h of incubation with the agonist, thus suggesting that itaconate is not a major mediator of TFEB and TFE3 under our experimental conditions.

**Figure 3.**
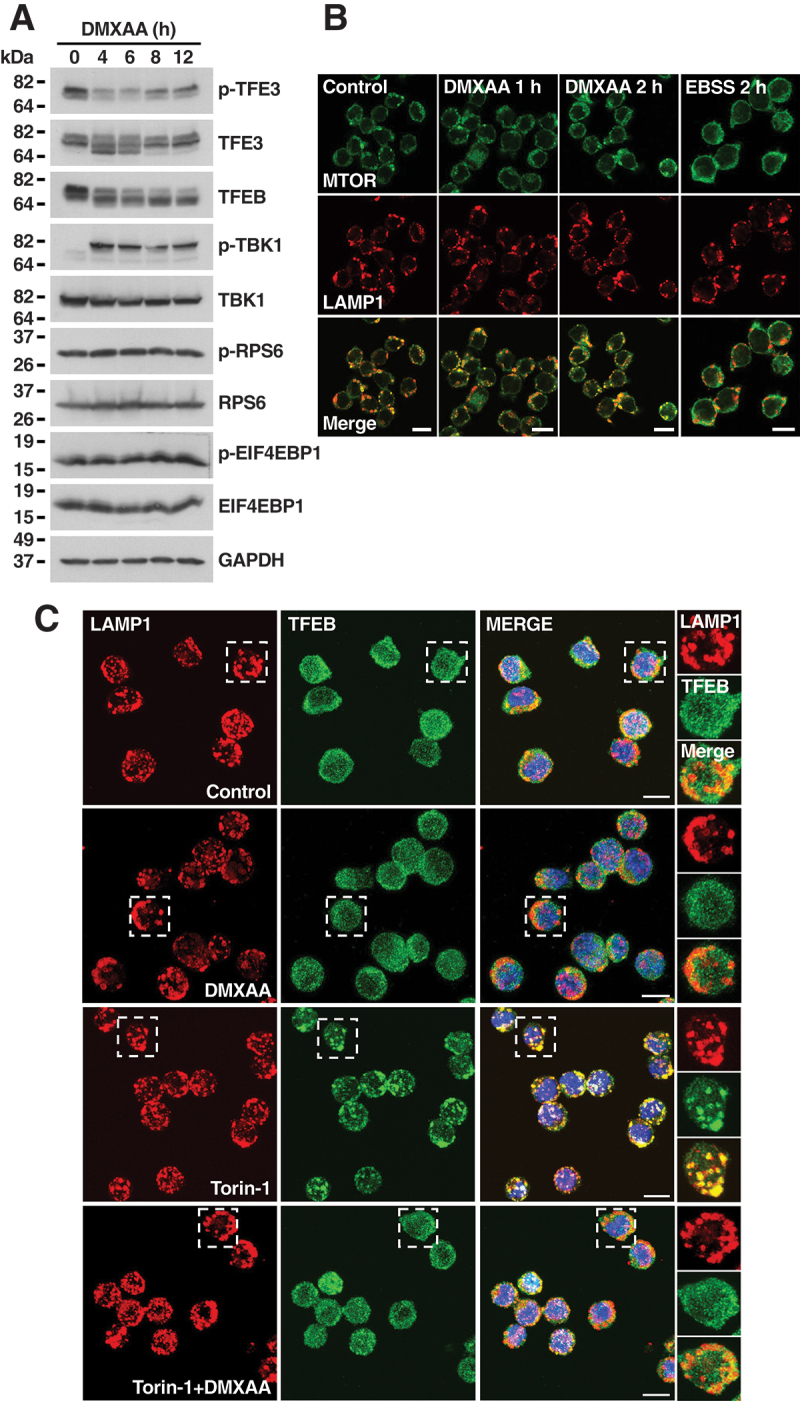
STING1-mediated activation of TFEB and TFE3 is independent of MTORC1 activity. (A) Representative immunoblot showing expression levels of p-TFE3 (S321), TFE3, TFEB, p-TBK1, TBK1, p-RPS6, RPS6, p-EIF4EBP1, and EIF4EBP1 in iBMDM cells treated with DMXAA (5 µg/ml) for 4, 6, 8, and 12 h. All immunoblots are representative of three independent experiments. (B) Representative immunofluorescence confocal microscopy of iBMDM cells showing the subcellular distribution of MTORC1 (green) and LAMP1 (red) in response to treatments with 10 µg/ml DMXAA or EBSS for 2 h. Scale bars: 10 μm. (C) Representative immunofluorescence confocal microscopy of iBMDM cells showing the subcellular distribution of TFEB (green) and LAMP1 (red) in response to treatments with 250 nM torin-1 and 10 µg/ml DMXAA for 1 h. Insets represent 2.42X magnification for control, 2.34X magnification for torin-1 and 2.5X magnification for torin-1+DMXAA. Scale bars: 10 μm.

Inactivation of MTORC1, the kinase that mediates phosphorylation of TFEB and TFE3 at serine 211 and serine 321, respectively, to induce cytosolic retention, is another important mechanism of TFEB and TFE3 activation [33–36]. We monitored MTORC1 activity in response to STING1 activation by measuring the phosphorylation levels of RPS6 and EIF4EBP1, two well-known MTORC1 substrates. As seen in Figures 3A, S3A, and S3B, phosphorylation of RPS6 and EIF4EBP1 remained constant during DMXAA treatment, indicating that MTORC1 is not inhibited by the agonist. Furthermore, association of MTORC1 with lysosomes, was not affected by DMXAA (Figure 3B). In contrast, inactivation of MTORC1 by starvation resulted in its redistribution to the cytosol, as previously described in other cell types (Figure 3B) [37].

Interaction of TFEB and TFE3 with RRAG GTPases is critical for the recruitment of the transcription factors to lysosomal membranes and their subsequent MTORC1-mediated phosphorylation [36,38,39]. Conformational changes of RRAG GTPases are sufficient to cause TFEB and TFE3 dephosphorylation and translocation to the nucleus [40,41]. To monitor interaction of TFEB and TFE3 with RRAGs, we incubated cells with torin-1, a treatment that increases RRAGs-mediated recruitment of the transcription factors to lysosomes [38] (Figure 3C and S3C). Interestingly, DMXAA prevented TFEB lysosomal accumulation even in the presence of torin-1 (Figures 3C and S3C), suggesting that STING1 activation alters RRAGs conformation, thus preventing TFEB and TFE3 cytosolic retention.

Finally, we tested the contribution of components of the STING1 signaling pathway. Upon translocation from the ER to the Golgi, STING1 recruits and activates the serine-threonine kinases TBK1 and IKBKE/IKKε, which in turn promote production of interferon and inflammatory cytokines [7]. Notably, we found that activation of TFEB and TFE3 in response to DMXAA was not inhibited in *tbk1* KO, *ikbke* KO, or *tbk1 ikbke* double-knockout (DKO) iBMDMs (Figure S3D). These results indicate STING1-mediated TFEB and TFE3 activation does not require TBK1 or IKBKE/IKKε signaling.

### TFEB binds to the promoter of lysosomal and immune genes in activated iBMDMs

**Figure 4.**
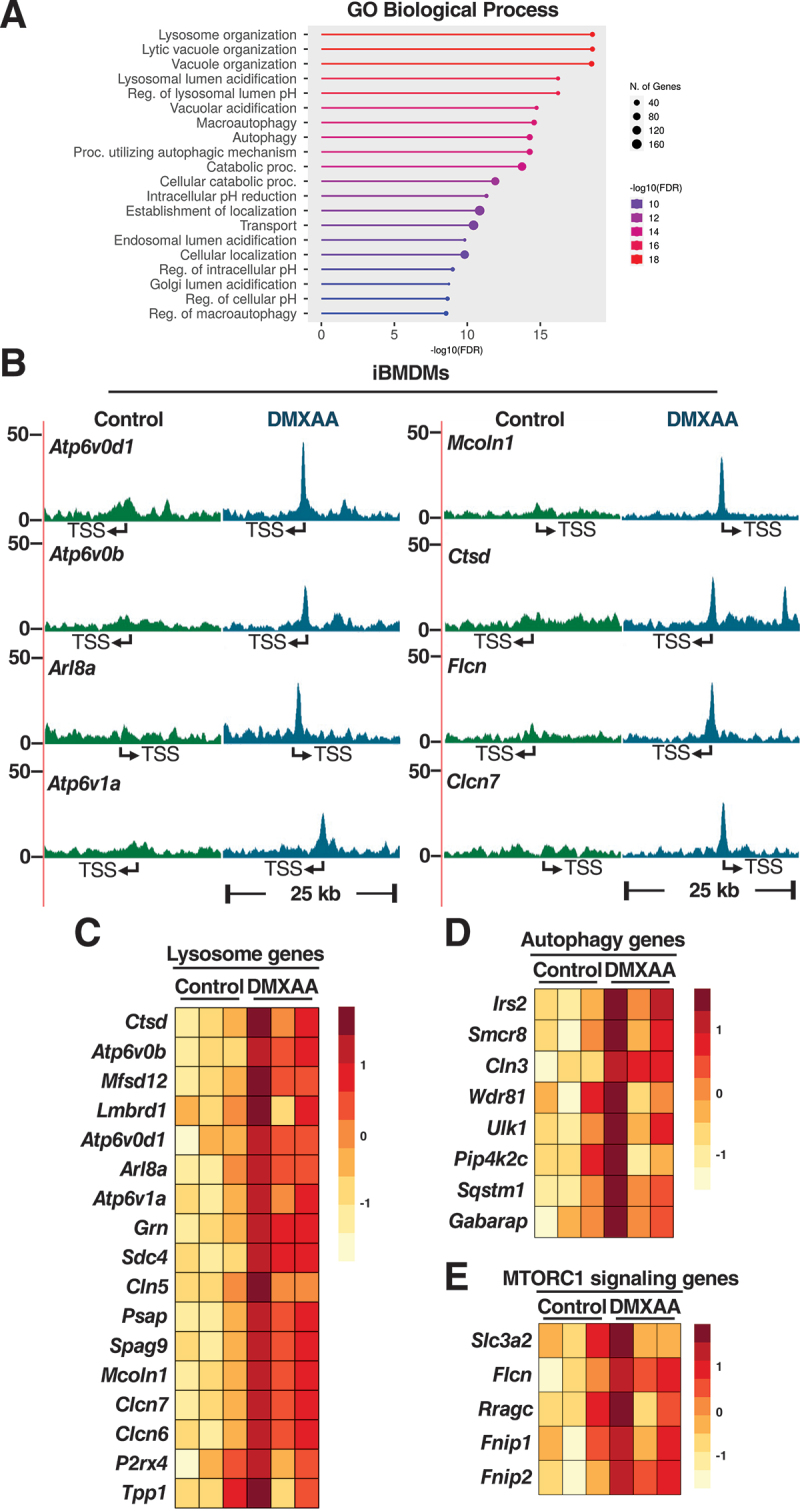
TFEB binds to the promoter of lysosomal and immune genes in STING1-activated iBMDMs. (A) Gene Ontology (GO) analysis of biological processes for genes identified from chromatin immunoprecipitation sequencing (ChIP-seq) of endogenous TFEB in iBMDMs treated with DMXAA (10 µg/ml) for 2 h. Circle size represents the number of genes, while the color scale indicates the -log10 of the false discovery rate (FDR). (B) ChIP-seq profile showing endogenous TFEB binding regions in the promoters of *Atp6v0d1*, *Atp6v0b*, *Arl8a*, *Atp6v1a*, *Mcoln1*, *Ctsd*, *Flcn*, and *Clcn7* in iBMDM cells treated with DMXAA (10 µg/ml) for 2 h. The transcription start site (TSS) is indicated. (C-E) Heatmaps of differentially expressed TFEB target genes related to lysosome function (C), autophagy (D), and MTORC1 signaling (E).

TFEB and TFE3 can modulate expression of hundreds of genes in response to different stress conditions, including many implicated in lysosomal, autophagic, metabolic, antioxidant, and immune processes [42]. To better understand the specific contribution of these transcription factors to the STING1-mediated cellular transcriptional response, we performed chromatin immunoprecipitation sequencing (ChIP-seq) analysis in iBMDMs treated with DMXAA for 2 h and assessed promoter occupancy by endogenous TFEB. We identified a total of 558 TFEB binding sites in the promoter region (from position −3,000 to 3,000). Correlative analysis between our ChIP-seq and RNA-seq data showed that the expression of 336 of these target genes was significantly increased upon STING1 activation (Table S3). Given the well-known role of TFEB in lysosomal biogenesis and autophagy, it was not surprising to find that GO analysis of TFEB target genes using Biological Processes and Cellular Compartment mainly identified categories related to lysosome organization and acidification, as well as autophagy regulation (Figures 4A and S4A). Robust TFEB binding peaks were observed in the proximal promoter region of many important lysosomal regulators, such as *Mcoln1*, *Ctsd*, *Clcn7*, *Arl8a*, and several subunits of the vacuolar-type proton-transporting ATPase (V-ATPase) complex, leading to their increased expression (Figure 4B,C). TFEB also showed binding to the promoter of several key modulators of autophagic (*Wdr81*, *Ulk1*, *Sqstm1*, *Gabarap*) and MTORC1 signaling (*Flcn*, *Fnip1*, *Fnip2*, *Rragc*) (Figure 4B,D,).

We recently showed that TFE3 directly upregulates expression of multiple immune regulators in response to beta-coronavirus infection [24]. Likewise, our ChIP-seq analysis revealed binding of endogenous TFEB to the promoter of immune genes in response to DMXAA (Table S3). The binding peaks corresponding to some of these genes, are shown in Figure S4B as an example. Furthermore, the expression levels of most of the TFEB immune targets was increased following STING1 activation (Figure S4C). Altogether, our data indicate that TFEB contributes to STING1-mediated responses by modulating transcription of lysosomal, autophagic, and immune genes.

### STING1-induced lysosomal biogenesis and autophagy are inhibited in the absence of TFEB and TFE3

**Figure 5.**
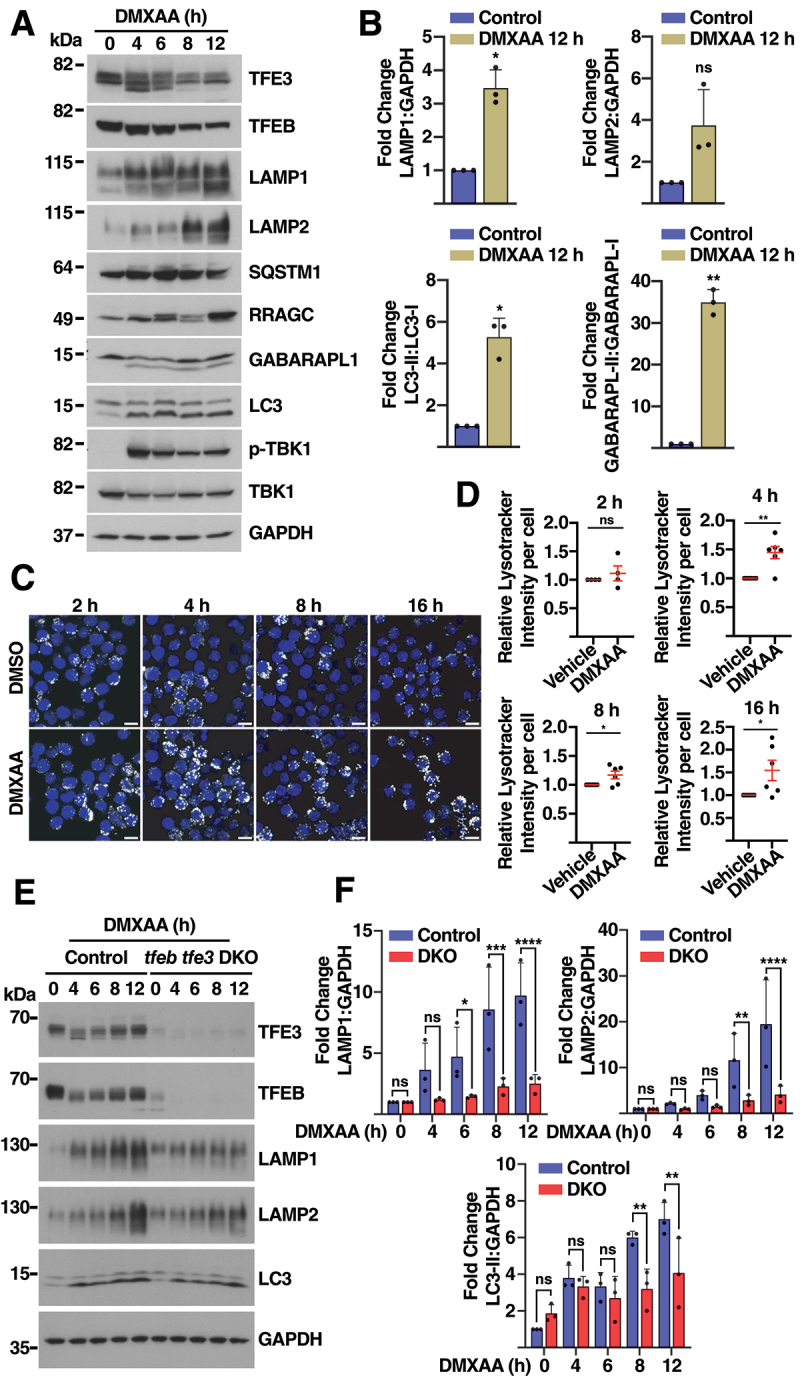
STING1-mediated activation of TFEB and TFE3 promotes autophagy and lysosomal biogenesis. (A) Representative immunoblot showing expression levels of TFE3, TFEB, LAMP1, LAMP2, SQSTM1, RRAGC, GABARAPL1, LC3, p-TBK1, and TBK1 in iBMDM treated with DMXAA (5 µg/ml) for 4, 6, 8, and 12 h. All immunoblots are representative of three independent experiments. (B) Quantification from immunoblots in (A) of total protein levels showing LAMP1:GAPDH, LAMP2:GAPDH, LC3-II:LC3-I, and GABARAPL-II:GABARAPL-I ratios at 12 h of treatment with DMXAA (*n* = 3). (C) Representative confocal images of iBMDM treated with DMXAA (5 µg/ml) for the indicated times and then incubated with LysoTracker DN-22 for 15 min. Scale bars: 10 μm. (D) Quantification of relative LysoTracker intensity per cell at 4, 6, 8, and 16 h post-treatment with DMXAA (5 µg/ml) (*n* = 4). Data are presented as mean ± SEM. (unpaired Student’s t‐test) (E) Representative immunoblot showing expression levels of TFE3, TFEB, LAMP1, LAMP2, and LC3 in control and TFEB- and TFE3-depleted iBMDM incubated with DMXAA (5 µg/ml) for 4, 6, 8, and 12 h (*n* = 3). (F) Quantification from immunoblots in (E) of total protein levels showing LAMP1:GAPDH, LAMP2:GAPDH, LC3-II:GAPDH. Data in (B) and (D) are presented as mean ± SD. (ns) not significant, (*) *p* < 0.05, (***) *p* < 0.001, and (****) *p* < 0.0001 (unpaired Student’s t‐test).

As shown in Figure 1A, treatment of iBMDMs with 10 μg/ml DMXAA caused a rapid and robust response; however, the activation was attenuated at 6 h treatment, presumably due to the delivery of STING1 to lysosomes and subsequent degradation. To better assess the effect of the STING1 pathway in the autophagic/lysosomal pathway, we treated iBMDMs with 5 μg/ml DMXAA for longer periods of time. This resulted in prolonged STING1 stimulation, as assessed by measuring TBK1 phosphorylation, as well as TFEB and TFE3 activation (Figure 5A). Under these conditions we observed substantial and persistent autophagy induction, as well as significant increase in the protein levels of different lysosomal proteins (Figure 5A,B).

Our RNA-seq analysis revealed STING1-mediated upregulation of several subunits of the V-ATPase complex, suggesting a potential increase in lysosomal acidification. To test this, DMXAA-treated iBMDMs were incubated with LysoTracker for 15 min, and then immediately imaged by confocal microscopy. Interestingly, we observed a significant increase in lysosomal acidification in DMXAA-treated cells when compared to untreated controls (Figure 5C,D). These data indicate that STING1 enhances the degradative capability of macrophages by increasing the number and activity of lysosomes.

To further investigate the participation of TFEB and TFE3 in STING1 signaling, we depleted both transcription factors in iBMDMs. Although we were unable to achieve a complete depletion, we did observe a reduction of over 70% in the levels of endogenous TFEB and TFE3 (Figure S5A and S5B). Simultaneous TFEB and TFE3 depletion did not affect early STING1 signaling, as determined by the comparable levels of active STING1 and TBK1 in control and knockdown cells, although the levels of active IRF3 were slightly augmented in TFEB- and TFE3-depleted iBMDMs (Figure S5A, S5C-E). Similar results were obtained in ARPE-19 cells in response to the human STING1 agonist diABZI (Figure S5F-H). However, the DMXAA-induced increase in LC3 lipidation, as well as LAMP1 and LAMP2 levels, were significantly diminished in TFEB- and TFE3-depleted cells (Figure 5E,F), corroborating the contribution of these transcription factors to STING1-mediated upregulation of autophagy and lysosomal biogenesis.

### Immune response alterations in TFEB- and TFE3-depleted iBMDMs

**Figure 6.**
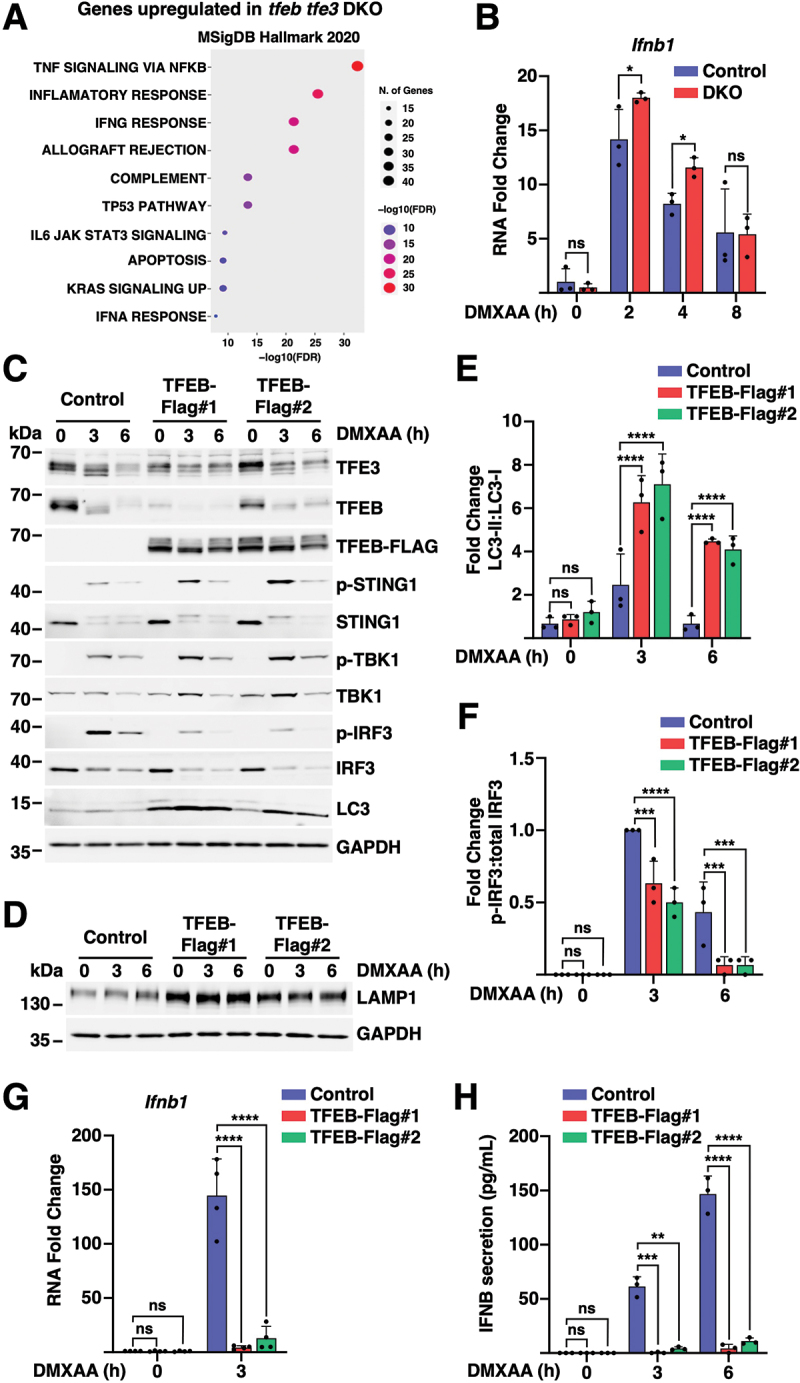
TFEB and TFE3 modulate interferon production by controlling levels of active IRF3. (A) Gene Ontology (GO) analysis of genes showing increased expression in TFEB and TFE3 knockdowns versus control cells in response to treatment with DMXAA (10 µg/ml) for 3 h. (B) Relative quantitative RT-PCR analysis of *Ifnb1* mRNA expression in iBMDM control and ***tfeb tfe3*** DKO cells treated with DMXAA (5 µg/ml) for 2, 4, and 8 h (*n* = 3). (C, D) Representative immunoblots showing expression levels of the indicated proteins in control iBMDMs and two TFEB-Flag expressing clones following treatment with DMXAA (10 µg/ml) for 3 and 6 h (*n* = 3). (E, F) Quantification from immunoblots in (D) showing of LC3-II:LC3-I (E) and p-IRF3:IRF3 (F) ratios (*n* = 3). (G) Relative quantitative RT-PCR analysis of *Ifnb1* mRNA expression in iBMDM control cells and TFEB-Flag expressing clones treated with DMXAA (10 µg/ml) for 3 and 6 h (*n* = 3). (H) ELISA analysis of mouse IFNB concentration in the supernatant of iBMDM control cells and TFEB-Flag expressing clones treated with DMXAA (10 µg/ml) for 3 and 6 h (*n* = 3). Data in (B), (E), and (F-H) are presented as mean ± SD. (ns) not significant, (*) *p* < 0.05, (***) *p* < 0.001, and (****) *p* < 0.0001 (two-way ANOVA).

Next, we performed RNA-seq analysis to compare global changes in gene expression between control and TFEB- and TFE3-depleted iBMDMs following treatment with DMXAA for 3 h (Figure S6A and Table S4). Ontological analysis confirmed that TFEB and TFE3 deficiency resulted in downregulation of lysosomal, autophagic, and MTORC1 signaling pathways (Figure S6B-D and Table S5). More unexpected was the increased expression of immune genes observed in TFEB/TFE3 knockdown cells. Whereas several of the immune TFEB targets identified in our ChIP-seq analysis were downregulated, including *Ccl3*, *Ccl4*, *Havcr2*, *Plau*, *Bhlhe40*, *Per1*, *C3ar1*, and *Sat1*, analysis of MSigDB Hallmark pathways revealed an increase in several immune categories related to inflammatory and interferon response, as well as apoptosis (Figure 6A and Table S6). Consistently, we found increased interferon production in TFEB- and TFE3-depleted iBMDMs (Figure 6B).

We reasoned that TFEB and TFE3 may limit or attenuate STING1 signaling. To further investigate this hypothesis, we used retroviral expression to generate iBMDM clones stably expressing human TFEB-Flag. Interestingly, whereas DMXAA treatment induced degradation of endogenous TFEB and TFE3, the levels of TFEB-Flag remained elevated (Figure 6C). This resulted in a much stronger induction of autophagy and lysosomal biogenesis (Figure 6C–E). Similar results were observed in two different clones. These data were further confirmed by qPCR. Expression of lysosomal genes was elevated in TFEB-Flag clones even in non-stimulated conditions but further increased in response to DMXAA (Figure S6E). Given the important role played by autophagy in IRF3 inactivation and inhibition of interferon response [43–45], we next assessed STING1 signaling. TFEB-Flag expression did not prevent efficient STING1 or TBK1 activation (Figures 6C and S6F); however, phospho-IRF3 levels were significantly reduced (Figure 6C,F). Accordingly, interferon expression and secretion were almost completely abolished in TFEB-overexpressing cells (Figure 6G,H). Furthermore, induction of several interferon-induced genes, such as *Socs1*, *Ifit1*, and *Isg15*, was strongly attenuated by recombinant TFEB (Figure S7A). Of note, the increased DMXAA-induced IRF3 degradation observed in TFEB-Flag clones was partially rescued by treatment with bafilomycin A_1_ (Figure S6G). These data indicate that robust and persistent autophagy activation in TFEB-overexpressing cells results in decreased IRF3 activation and interferon production, revealing an unexpected role of TFEB in STING1 signaling attenuation.

### TFEB and TFE3 promote STING1 signaling attenuation and cell survival

**Figure 7.**
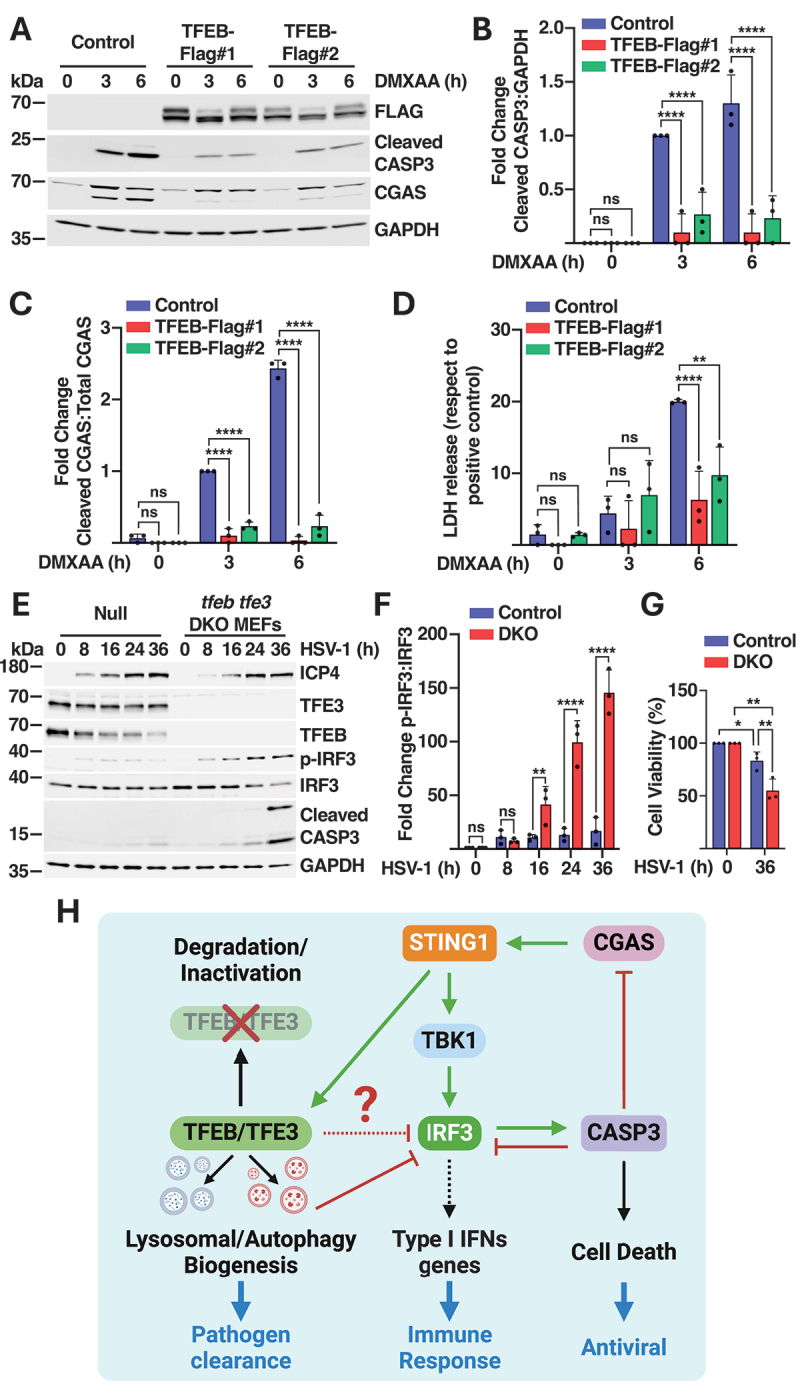
TFEB and TFE3 prevent cell death in response to STING1 activation. (A) Representative immunoblot showing expression levels of TFEB-FLAG, cleaved CASP3, and CGAS in control and TFEB-Flag expressing iBMDMs, treated with DMXAA (10 µg/ml) for 3 h and 6 h (*n* = 3). (B, C) Quantification of total protein levels from immunoblots in (A) showing ratios of cleaved CASP3:GAPDH (B) and cleaved CGAS:total CGAS (C) as fold increases (*n* = 3). (D) Quantification of LDH levels released into the extracellular media of control and TFEB-Flag expressing iBMDMs treated with DMXAA (10 µg/ml) for 3 and 6 h (*n* = 3). (E) Representative immunoblot showing expression levels of ICP4, TFE3, TFEB, p-IRF3, IRF3, cleaved CASP3, and GAPDH in MEFs cells infected with HSV-1 (MOI 2.5) for 8, 16, 24, and 36 h (*n* = 3). (F) Quantification of total protein levels showing the ratios of p-IRF3:IRF3 at 8 h, 16 h, 24 h, and 36 h as fold increases from immunoblots in (E) (*n* = 3). (G) Cell viability of control and ***tfeb tfe3*** DKO MEFs infected with HSV-1 at an MOI of 2.5 for 36 h (*n* = 3). (H) Model depicting the proposed role of TFEB and TFE3 in the regulation of the STING1 pathway. Data in (B-D) and (F, G) are presented as mean ± SD. (ns) not significant, (*) *p* < 0.05, (**) *p* < 0.01 (***) *p* < 0.001, and (****) *p* < 0.0001 (two-way ANOVA).

Several recent reports have shown that STING1-mediated cell death is another important component of the antiviral response. Phosphorylation of IRF3 by active STING1 induces formation of a complex between IRF3 and BAX, resulting in activation of caspases and apoptosis [46]. Caspase activation also serves to attenuate STING1 signaling by promoting cleavage of CGAS and IRF3 [47]. In agreement with these observations, we found that the lack of IRF3 phosphorylation in DMXAA-treated *tbk1 ikbke* DKO cells prevented CASP3 (caspase 3) cleavage, further confirming the relevance of the TBK1-IRF3-CASP3 axis (Figure S7B and S7C). We then hypothesized that the increased IRF3 degradation observed in TFEB-overexpressing iBMDMs might lead to reduced caspase activation. Consistent with this idea, we observed significantly lower levels of active CASP3, as well as reduced CGAS cleavage in our two TFEB-FLAG expressing clones (Figure 7A–C). To assess the impact on cell viability, we measured LDH levels in the supernatant. LDH is a cytosolic enzyme and its presence in the extracellular media is indicative of cell rupture. As expected, we found that TFEB overexpression significantly increased cell survival (Figure 7D). Conversely, CASP3 activation and cell death were increased in TFEB- and TFE3-depleted iBMDMs (Figure S7D-F).

To further expand these observations, we infected control and *tfeb tfe3* double-knockout (DKO) MEFs with herpes simplex virus-1 (HSV-1). The expression of the viral protein ICP4 was measured to confirm HSV-1 infection. In agreement with our results in iBMDMs, we observed significantly higher levels of phospho-IRF3 and cleaved CASP3 in *tfeb tfe3* DKO in response to viral infection (Figures 7E,F, and S7G), as well as reduced cell viability (Figure 7G). Altogether, our data suggest that TFEB and TFE3 play a critical role in the modulation of the innate immune response. Whereas these transcription factors may increase the cell degradative capabilities by promoting efficient pathogen clearance, they also prevent excessive STING1 signaling, thus favoring survival (Figure 7H).

## Discussion

STING1 plays a critical role in the production of type I interferons and inflammatory cytokines in response to DNA derived from pathogens or damaged host cells. In this study we show that the transcription factors TFEB and TFE3 function as negative regulators of STING1-mediated innate antiviral immunity by attenuating type I IFN signaling. STING1 activation induced TFEB and TFE3 nuclear translocation in iBMDMs, resulting in increased autophagy, lysosomal biogenesis, and lysosomal acidification. This increases the degradative capabilities of macrophages, facilitating the removal of virus and bacteria. It is well established that STING1 can promote canonical autophagy through inactivation of MTORC1 and activation of BECN1 [15,48], as well as ATG5-dependent non-canonical autophagy [49,50]. Our data expand these observations by showing that STING1 also regulates transcriptional programs leading to the enhancement of the autophagosome/lysosome pathway.

Negative regulation of immune pathways is essential to resolve immune responses and prevent excess inflammation. Selective autophagy is key to suppress activation of type I IFN signaling by targeting crucial immune factors, such as STING1, CGAS and IRF3. Depletion of TFEB and TFE3 in iBMDMs diminished the autophagy induction induced by STING1 agonists. Conversely, TFEB overexpression resulted in a much more robust autophagy stimulation, resulting in significantly lower levels of active IRF3 and an almost complete absence of IFNB/IFNβ production. This suggests that TFEB and TFE3 play an important role in modulating the crosstalk between autophagy and IFN signaling, although we cannot rule out their involvement in additional mechanisms underlying the precise control of IRF3 activation.

Apoptotic caspases also contribute to maintaining immune homeostasis. Activation of CASP3 by active IRF3 and consequent cell death is an important component of the host defense against viruses. At the same time CASP3 cleaves CGAS and IRF3 to suppress immune activation. By regulating the levels of active IRF3, TFEB and TFE3 modulate not just interferon production, but also cell viability. As such, we found that TFEB overexpression strongly inhibited CASP3 activation and promoted cell survival, while TFEB and TFE3 depletion resulted in increased cell death in iBMDMs. Absence of TFEB and TFE3 also resulted in increased caspase activation and cell death in MEFs infected with HSV-1, further demonstrating the important role played by these transcription factors in antiviral response. Since STING1 activation may lead to cell death through a variety of mechanism besides apoptosis [5], future studies should address the potential contribution of TFEB and TFE3 to STING1-mediated necroptosis, pyroptosis and ferroptosis.

TFEB and TFE3 can be activated via different mechanisms. Inactivation of MTORC1 during nutrient deprivation [33–36] or activation of specific phosphatases, such as PP2A and calcineurin, in response to starvation, oxidative stress, and MHV infection [24,51,52] promote TFEB and TFE3 dephosphorylation and translocation to the nucleus. The role of the FLCN-FNIP complex is also becoming increasingly relevant. By promoting GDP-loading of RRAGC [53], FLCN-FNIP facilitates interaction of TFEB and TFE3 with RRAGs, resulting in retention of the transcription factors in the cytosol [38,54]. GABARAP lipidation following lysosomal damage causes FLCN sequestration and inhibition of its GAP activity, resulting in TFEB and TFE3 activation [40]. LC3 and GABARAP lipidation also contribute to TFEB activation in response to kidney injury and pathogen infection [55,56]. Furthermore, since the channel activity of STING1 promotes LC3 and GABARAP lipidation of membranes [10], it is likely that STING1 activation inhibits FLCN-FNIP function. These observations are consistent with our results showing that DMXAA prevented the interaction between TFEB and RRAGs in intact macrophages, further suggesting a broader role of mammalian Atg8s in TFEB-mediated immune response.

During the preparation of this manuscript, two studies were published showing that the channel activity of STING1 is required for TFEB activation in fibroblasts, indicating that the STING1-GABARAP-FLCN pathway also operates in nonimmune cells [57,58]. Consistent with our work, these studies reported TFEB-dependent transcriptional upregulation of lysosomal genes following STING1 activation, which resulted in increased degradative capability and survival. However, while these studies suggest that the TFEB and TFE3 activation and interferon production may be part of two separate pathways, with the STING1-TFEB-TFE3 axis being part of a primordial antiviral response, our data show that both pathways converge due to the ability of TFEB and TFE3 to modulate IRF3 activation and interferon production.

Our study also reveals the strict regulation of TFEB and TFE3 activation in macrophages. By inducing the transient activation or degradation of these transcription factors, cells may further adjust the strength of the inflammatory response. The decrease in TFEB and TFE3 levels observed in iBMDMs may be part of a negative feed-back loop, as we detected DMXAA-induced binding of TFEB to the promoter of BHLHE40 and BHLHE41, two well-known TFEB and TFE3 transcriptional repressors [59]; however, we cannot rule out the presence of additional mechanisms that directly promote protein degradation. One would predict that pathogens may benefit from targeting TFEB and TFE3 stability. In fact, it has been described that both, Coxsackievirus B3 (CVB3) and beta-coronavirus induce their degradation. Thus, CVB3 proteinase 3B cleaves TFEB to suppress its transcriptional activity [60], while beta-coronavirus induces DCAF7-dependent TFEB and TFE3 protein degradation [24,61]. In some cases, the regulation is more complex. Some viruses may benefit from inducing TFEB and TFE3 activation at early infection times as a way to prevent cell death and enhance production of autophagosomal membranes that they require for replication. At later infection times, however, TFEB and TFE3 degradation or inactivation may avert autophagy-mediated clearance of assembled viral particles and even facilitate virus dissemination by inducing cell death. This type of bimodal regulation has been previously reported for human immunodeficiency virus, which induces early TFEB activation followed by Nef-mediated hyperactivation of MTORC1 and consequent TFEB cytosolic sequestration [23]. It is also important to consider that dysregulation of DNA-sensing pathways greatly contributes to a variety of diseases, including autoimmunity and cancer [1,62]. Therefore, modulation of TFEB and TFE3 levels and activation may represent an important therapeutic target for treatment of inflammatory disorders.

In summary, we have identified a novel and important role of TFEB and TFE3 in fine-tuning STING1 immune responses by balancing interferon production, signaling attenuation, and cell survival.

## Materials and methods

### Cell lines cultures

Reagent information not listed here is provided in Table 1. iBMDMs were cultured in RPMI 1640, RAW 264.7 and mouse embryonic fibroblasts (MEFs) Control and *tfeb tfe3* DKO cells [63] were cultured in DMEM, and ARPE-19 cells were cultured in DMEM: Ham’s F12. Media was supplemented with high glucose, GlutaMAX™, 10% (v:v) FBS and 1% (v:v) penicillin-streptomycin, and cultures were maintained at 37°C in 5% CO_2_.

**Table 1.** Reagents and tools.

Reagent/Resource	Reference or Source	Identifier or Catalog Number
**Experimental Models**		
Immortalized Mouse Macrophage Cells (iBMDM) (BMA3.1A7)	abm	T0673
RAW 264.7	ATCC	TIB-71
ARPE-19	ATCC	CRL-2302
Mouse embryonic fibroblasts CRISPR *tfeb tfe3* DKO	[63]	N/A
WT immortalized bone marrow-derived macrophages (iBMDMs)	[7]	N/A
*tbk1* KO immortalized bone marrow-derived macrophages (iBMDMs)	[7]	N/A
*ikbke* KO immortalized bone marrow-derived macrophages (iBMDMs)	[7]	N/A
*tbk1 ikbke* DKO immortalized bone marrow-derived macrophages (iBMDMs)	[7]	N/A
iBMDM TFEB-Flag#1	This paper	N/A
iBMDM TFEB-Flag#2	This paper	N/A
**Antibodies**		
GOLGA2/GM130	BD Biosciences	610822
STING1/TMEM173	Proteintech Group, Inc.	19851–1-AP
STING1 (D2P2F)	Cell Signaling Technology	13647
Phospho-STING1 (Ser365)	Cell Signaling Technology	72971
TBK1/NAK (D1B4)	Cell Signaling Technology	3504
Phospho-TBK1/NAK (Ser172) (D52C2)	Cell Signaling Technology	5483
IRF3 (D83B9)	Cell Signaling Technology	4302
Phospho-IRF3 (Ser396) (4D4G)	Cell Signaling Technology	4947
Phospho-RELA/NFKB p65 (Ser536) (93H1)	Cell Signaling Technology	3033
RELA/NFKB p65 (D14E12)	Cell Signaling Technology	8242
TFEB	Bethyl Laboratories, Inc	A303-673A
p-TFE3 (S321)	[63]	N/A
TFE3	Sigma Aldrich	SAB4200824-100 U
TFE3	Cell Signaling Technology	14779
TBP (D5C9H)	Cell Signaling Technology	44059
Goat anti-Rabbit IgG (Heavy Chain), Superclonal Recombinant Secondary Antibody, Alexa Fluor 488	Thermo Fisher Scientific Inc	A27034
Goat anti-Rabbit IgG (Heavy Chain), Superclonal Recombinant Secondary Antibody, Alexa Fluor 555	Thermo Fisher Scientific Inc	A27039
GAPDH	Thermo Fisher Scientific Inc	AM4300
Anti-rabbit IgG, HRP-linked Antibody	Cell Signaling Technology	7074
Anti-mouse IgG, HRP-linked Antibody	Cell Signaling Technology	7076
Anti-rat IgG, HRP-linked Antibody	Cell Signaling Technology	7077
LAMP1	DSHB	1D4B
LAMP2	Cell Signaling Technology	34141
FLAG	Sigma Aldrich	F3165
ACOD1/IRG1	Cell Signaling Technology	19857
EIF4EBP1 (53H11)	Cell Signaling Technology	9644
Phospho-EIF4EBP1 (Ser65)	Cell Signaling Technology	9451
RPS6 Ribosomal Protein (5G10)	Cell Signaling Technology	2217
Phospho-RPS6 Ribosomal Protein (Ser240/244) (D68F8)	Cell Signaling Technology	5364
LC3B (E5Q2K)	Cell Signaling Technology	83506
SQSTM1/p62	Cell Signaling Technology	5114
GABARAPL1 (D5R9Y)	Cell Signaling Technology	26632
RRAGC (D8H5)	Cell Signaling Technology	9480
IKBKE/IKKε (D61F9)	Cell Signaling Technology	3416
ICP4	Abcam	ab6514
**Viruses and plasmids**		
Human herpesvirus 1	ATCC	VR-1493
pMXs-Puro	Cell Biolabs	RTV-012
**Oligonucleotides and other sequence-based reagents**		
Alt-R® CRISPR-Cas9 Negative Control crRNA #1	IDT	1072544
Alt-R CRISPR-Cas9 crRNA, Mm.Cas9.TFE3.1.AE	IDT	Mm.Cas9.TFE3.1.AE
Alt-R CRISPR-Cas9 crRNA, Mm.Cas9.TFEB.1.AD TFEB	IDT	Mm.Cas9.TFEB.1.AD TFEB
Alt-R® CRISPR-Cas9 tracrRNA, ATTO™ 550	IDT	1075927
ON-TARGETplus Non-targeting Control Pool	Horizon Discovery	D-001810
ON-TARGETplus Human *TFEB* siRNA	Horizon Discovery	L-009798
ON-TARGETplus Human *TFE3* siRNA	Horizon Discovery	L-009363
qPCR Primers	This study	Table S7
**Chemicals, Enzymes and other reagents**		
Pierce™ IP Lysis Buffer	Thermo Fisher Scientific Inc	87788
Halt™ Protease and Phosphatase Inhibitor Cocktail (100X)	Thermo Fisher Scientific Inc	78440
NuPAGE™ LDS Sample Buffer (4X)	Thermo Fisher Scientific Inc	NP0007
NuPAGE™ Sample Reducing Agent (10X)	Thermo Fisher Scientific Inc	NP0009
Nitrocellulose/Filter Paper Sandwich, 0.2 μm, 8.3 × 7.3 cm	Thermo Fisher Scientific Inc	LC2000
DMXAA – Murine STING1 ligand – Xanthenone Analog	InvivoGen	tlrl-dmx
EBSS, calcium, magnesium, phenol red	Thermo Fisher Scientific Inc	24010043
Torin 1	Tocris	4247
LysoTracker™ Red DND-99	Thermo Fisher Scientific Inc	L7528
HCS CellMask Deep Red Stain	Thermo Fisher Scientific Inc	H32721
diABZI STING1 Agonist-3	Cayman Chemical Company	34082
Hoechst 33,342 Solution (20 mm)	Thermo Fisher Scientific Inc	62249
Alt-R™ S.p. Cas9 Nuclease V3	IDT	1081059
Opti-MEM™ I Reduced Serum Medium	Thermo Fisher Scientific Inc	31985062
Lipofectamine™ RNAiMAX Transfection Reagent	Thermo Fisher Scientific Inc	13778075
RPMI 1640 Medium, GlutaMAX™ Supplement	Thermo Fisher Scientific Inc	61870127
DMEM/F-12, GlutaMAX™ supplement	Thermo Fisher Scientific Inc	10565018
DMEM, high glucose, GlutaMAX™ Supplement, pyruvate	Thermo Fisher Scientific Inc	10569044
**Software**		
Fiji	https://imagej.net/Fiji	
QuantStudio 12K Software v1.5	https://www.perkinelmer.com/product/image- data-storage-and-analysis-system-columbus	
GraphPad Prism 10.3.1	https://www.graphpad.com/scientific-software/ prism/	
Columbus 2.9.1.532		
**Other**		
Cell culture microplate, 96 well, PS, F-Bottom	Greiner Bio-One	655090
Nunc™ Lab-Tek™ II Chambered Coverglass	Thermo Fisher Scientific Inc	155409
4D-Nucleofector® Core Unit	Lonza	AAF-1003B
4D-Nucleofector® X Unit	Lonza	AAF-1003X
Pierce™ BCA Protein Assay Kits	Thermo Fisher Scientific Inc	23225
RNeasy Mini Kit	Qiagen	74104
TruSeq Stranded mRNA Library kit	Illumina	20020594
PureLink™ RNA Mini Kit	Thermo Fisher Scientific Inc	12183018A
SuperScript™ III First-Strand Synthesis SuperMix for qRT-PCR	Thermo Fisher Scientific Inc	11752050
Fast SYBR™ Green Master Mix	Thermo Fisher Scientific Inc	4385612
Fast SYBR™ Green Master Mix	Thermo Fisher Scientific Inc	4385612
LumiKine™ Xpress MmIFNB/mIFN-ß 2.0	InvivoGen	luex-mifnbv2
LDH Cytotoxicity Assay Kit	Cell Signaling Technology	37291S
P3 Primary Cell 4D-Nucleofector™ X Kit S	Lonza	V4XP–3032
RealTime-Glo™ MT Cell Viability Assay	Promega	G9711

### Western blotting

Total lysates from iBMDMs were prepared by washing with cold 1X PBS (Thermo Fisher Scientific Inc., 70011044), followed by addition of RIPA Lysis Buffer (Thermo Fisher Scientific Inc., 89900) with Protease and Phosphatase Inhibitor Cocktail. The lysates were centrifuged at 14,000 × g for 30 min at 4°C. Proteins were separated by SDS-PAGE and transferred to nitrocellulose membranes. Membranes were blocked with 5% milk in TBS (Thermo Fisher Scientific Inc., J60877.K3), then incubated overnight with the primary antibody at 4°C. Afterward, membranes were incubated with HRP-conjugated secondary antibody (1:5000–1:10,000) at room temperature. Detection was performed using SuperSignal West Femto (Thermo Fisher Scientific Inc., 34096), and chemiluminescence was captured with the C300 Gel Imaging System (Azure Biosystems). Quantification was done by densitometry using ImageJ Fiji.

### Subcellular fractionation

Cells were harvested in resuspension buffer (10 mM HEPES, pH 7.4, 10 mM KCl, 1.5 mM MgCl_2_, 1.5 mM dithiothreitol) supplemented with protease and phosphatase inhibitors at a concentration of 6.5 × 10^6^ cells/ml. Cells were gently mixed and kept on ice for 10 min. Then, cells were lysed by the addition of 0.05% NP-40 (Thermo Fisher Scientific Inc., 85125) and gently mixing for 3 s. The lysate was then centrifugated at 800 × g for 10 min at 4°C. The supernatant was centrifuged at 13,500 × g for 15 min at 4°C. The resulting supernatant represents the cytosolic fraction. The 800 × g pellet was washed in resuspension buffer containing NP-40 followed by centrifugation at 800 × g for 10 min at 4°C. The resulting pellet was resuspended in nuclear extraction buffer (20 mM HEPES, pH 7.4, 400 mM NaCl, 1.5 mM MgCl_2_, 0.2 mM EDTA, 10% glycerol, 0.2% NP-40) supplemented with protease and phosphatase inhibitors and kept on ice for 30 min with intermittent vigorous vortexing. The supernatant obtained after centrifugation at 18,500 × g for 30 min at 4°C contained the nuclear fraction.

### Generation of iBMDM tfeb tfe3 double-knockout (DKO) cells

To generate *tfeb tfe3* DKO iBMDM cells, CRISPR-Cas9 technology was employed. Specific crRNAs targeting TFEB and TFE3, or a negative control, along with tracrRNA, were combined at a 1:1 ratio and heated to 95°C for 5 min to form the crRNA-tracrRNA complex. Cas9 protein (10 µg) and Duplex buffer were added and incubated at room temperature for 10 min. iBMDM cells (0.5 × 10^6^ cells/ml) were resuspended in 20 µL of P3 buffer and electroporated with the crRNA-tracrRNA-Cas9 complex using the 4D-Nucleofector (Lonza) and the CM137 program. Immediately after electroporation, prewarmed complete media was added. Cells were seeded in a P100 plate (Corning Incorporated 353003) and expanded after 3 days for subsequent experiments.

### Real-time PCR

Total RNA was isolated using the PureLink RNA Mini Kit. Reverse transcription was performed with SuperScript III First-Strand Synthesis SuperMix for qRT-PCR. Real-time PCR was conducted using Fast SYBR™ Green Master Mix on the QuantStudio 12K Flex real-time PCR system. Relative gene expression was normalized to *Gapdh*, and RNA expression was calculated using the 2^-ΔΔCt method.

### RNA-Seq

Wild-type (WT) iBMDMs and iBMDMs generated by CRISPR (Negative control and *tfeb tfe3* DKO) were incubated for 3 h with the murine agonist DMXAA (10 µg/ml). Total RNA was isolated using the Qiagen RNeasy Mini Kit. For each sample, 0.5 µg of total RNA was used to generate libraries with Illumina’s TruSeq Stranded mRNA Library Kit. Libraries were sequenced on the Illumina NextSeq 500 platform as paired-end 42-nt reads. Sequence data were analyzed using the STAR alignment and DESeq2 software pipeline.

### ChIP-seq

iBMDMs were incubated with DMXAA (10 µg/ml) for 2 h, then crosslinked with 1/10 volume of Formaldehyde Solution for 15 min at room temperature. Crosslinking was quenched with 0.5 volume of glycine (2.5 M). Cells were centrifuged at 800 × g for 10 min at 4°C, resuspended in cold PBS with 0.5% Igepal CA-630 (Thermo Fisher Scientific Inc., 9002-93-1) and 1 mm PMSF (Thermo Fisher Scientific Inc., 36978), and washed. After final centrifugation, the pellet was snap-frozen on dry ice and processed for ChIP-Seq by Active Motif, Inc. Anti-TFEB (Bethyl Laboratories, Inc., A303-673A) was used for immunoprecipitation.

### Measurement of cell death

Relative LDH levels were assessed in cell culture media. The media were first centrifuged at 500 × g for 5 min at room temperature, then analyzed in 96-well plates using the LDH Cytotoxicity Assay Kit.

### Cell viability assay

Cell viability was assessed using the Promega RealTime-Glo™ MT Cell Viability Assay, following the manufacturer’s instructions.

### High-throughput imaging

RAW 264.7 cells were seeded in 96-well plates and incubated with DMXAA (10 µg/ml). Cells were then fixed, permeabilized, and blocked. After overnight incubation with the primary antibody, cells were incubated with the secondary antibody. To stain the membrane and nucleus, HCS CellMask Deep Red and Hoechst 33342 were used. Images were acquired using the Cell Voyager (CV7000) at 20X magnification, capturing 16 images per well. Image analysis was performed with Columbus 2.9.1.532 software.

### siRNA transfection for TFEB and TFE3 knockdown

ON-TARGETplus Human siRNA targeting *TFEB* and *TFE3*, or the ON-TARGETplus Non-targeting Control Pool, was mixed with Opti-MEM I Reduced Serum Medium and Lipofectamine RNAiMAX Transfection Reagent. The mixture was then added to the cells, achieving a final siRNA concentration of 100 nM. After 72 h, the cells were used for treatment.

### Recombinant DNA plasmid

TFEB-FLAG retroviral expression vector was generated by PCR amplification of the full-length encoding sequence of human TFEB followed by in-frame cloning into EcoRI of pMXs-Puro vector (Cell Biolabs, R3101S) with a triple FLAG tag fused to the carboxy-termini of TFEB. Construct was confirmed by DNA sequencing.

### Retroviral particles production and target cell transduction

Plat-E cells (5 ×10^6^) in 10-cm plates were transfected with 15 μg of pMXs-TFEB-FLAG plasmid and 45 μl Fugene HD (Promega Corp., E2311). Forty-eight hours post transfection, the medium from the transfected Plat-E cells was collected and passed through a 0.22-μm filter. Three milliliters of filtered medium containing the viral particles were mixed with polybrene (10 μg/ml) and added to 0.2 × 10^6^ iBMDM cells in a 6-well plate. Sixteen hours post infection, the medium was replaced with fresh medium, and cells were allowed to recover for an additional 40 h. Antibiotic selection was performed with 2 μg/ml puromycin, and isolation of single cell clones from the selected pool was achieved using limiting dilution cloning.

### HSV-1 infection

MEF cells were seeded and cultured to over 90% confluence. Then cells were infected with HSV-1 at a MOI of 2.5 for 1 h in serum- and antibiotic-free DMEM. After infection, the cells were washed three times with PBS and then incubated in complete medium for the indicated times.

### Confocal microscopy

Cells were seeded on coverslips in 6-well plates. After treatment, they were washed with PBS and fixed in 4% paraformaldehyde for 20 min. Cells were then permeabilized with 0.2% Triton X-100 (Sigma Aldrich, T9284) for 10 min, followed by blocking with 3% BSA (Sigma Aldrich, A3294) in PBS for 1 h at room temperature. Next, cells were incubated with primary antibodies overnight at 4°C, followed by incubation with secondary antibodies for 1 h at room temperature. For live cell imaging, 0.3 × 10^6^ cells/ml were plated on 8-well Nunc™ Lab-Tek™ II Chambered Coverglass overnight. Fifteen min before imaging, cells were incubated with 50 nM LysoTracker Red DN-99, then washed three times with dye-free media. Cells were transferred to a prewarmed microscope stage and treated with DMXAA (5 µg/ml) or DMSO as a control. Imaging was performed using a Zeiss LSM 780 AxioObserver. LysoTracker and STING1 fluorescence intensity, TFE3 nuclear localization and LAMP1-positive puncta were quantified using ImageJ analysis software (NIH).

### Statistical analyses

Data were processed using Excel (Microsoft Corporation) and Prism (GraphPad Software) to generate bar charts and perform statistical analyses. Student’s t‐test or two‐way ANOVA and pairwise post‐tests were run for each dependent variable, as specified in each figure legend. *p < 0.05, **p < 0.01, ***p < 0.001, ****p < 0.0001. *p* > 0.05 was considered not significant (ns). Data are presented as mean ± SD.

## Supplementary Material

Table S3.xlsx

Table S7.xlsx

Table S4.xlsx

Table S2.xlsx

Table S6.xlsx

Table S5.xlsx

Table S1.xlsx

Supplementary Materials.docx

## Data Availability

All source and supporting data are available from the corresponding authors on reasonable request. RNA-sequencing and ChIP-sequencing data from this publication have been deposited in the NCBI GEO database and assigned the following identifier numbers: RNA-seq analysis of iBMDMs treated with DMXAA for 3 h: **GSE281221** https://www.ncbi.nlm.nih.gov/geo/query/acc.cgi?acc=GSE281221 RNA-seq analysis of control and TFEB- and TFE3-depleted iBMDMs treated with DMXAA for 3 h: **GSE281234** https://www.ncbi.nlm.nih.gov/geo/query/acc.cgi?acc=GSE281234 ChIP-seq analysis of iBMDMs treated with DMXAA for 2 h: **GSE281183** https://www.ncbi.nlm.nih.gov/geo/query/acc.cgi?acc=GSE281183
