# Supplementary material for: TFEB and TFE3 regulate STING1-dependent immune responses by controlling type I interferon signaling
Source: Autophagy. 2025 Apr 20;21(9):2028–45. doi: 10.1080/15548627.2025.2487036 (PMC12363505; doi:10.1080/15548627.2025.2487036)
Supplement: Supplementary Materials.docx [file KAUP_A_2487036_SM9168.docx]

Supplementary Materials for

**TFEB and TFE3 regulate STING1-dependent immune responses by controlling type I interferon signaling**

Pablo Tapia *et al.*

Corresponding author: Rosa Puertollano, [puertolr@mail.nih.gov](mailto:puertolr@mail.nih.gov)

**The file includes:**

**Supplementary Figures S1 to S7**

**Other Supplementary Material for this manuscript includes the following:**

**Tables S1-S7 (provided as Excel files)**

**Figure S1.** STING1-dependent immune response. (**A**) Relative quantitative RT-PCR analysis of *Ifnb1* mRNA expression in iBMDM cells treated with DMXAA (10 µg/ml) for 2, 3, and 6 h (n = 4). (**B**) ELISA analysis of mouse IFNB levels in the supernatant of iBMDM cells at 2, 3, and 6 h after treatment with DMXAA (10 µg/mL), shown as fold increase relative to the 3 h treatment (n = 4). (**C**) Principal-component analysis (PCA) of genes with q-value < 0.05 shows distinct clustering of control and DMXAA-treated iBMDM cells at the 3 h time point (n = 3). (**D**) KEGG pathway enrichment analysis of differentially expressed genes in DMXAA-treated cells. Circle size represents the number of genes, while the color scale indicates the -log10 of the false discovery rate (FDR) (n = 3). (**E-I**) Relative quantitative RT-PCR analysis of mRNA expression for *Ccl2* (**E**), *Ccl3* (**F**), *Ccl4* (**G**), *Cxcl10* (**H**), and *Ifit1*. (**I**) in iBMDM cells treated with DMXAA (10 µg/mL) for 2, 3, and 6 h (n = 4). Data in (A-B) and (E-I), data are presented as mean ± SD. (ns) not significant, (*) p<0.05, (***) p<0.001, and (****) p<0.0001 (One-way ANOVA followed by Dunnett’s multiple comparison post-test).

**Figure S2.** STING1-dependent activation of TFEB and TFE3 in RAW264.7. (**A**) Representative immunofluorescence images of STING1 (green), GOLGA2/GM130 (red), and nuclei (blue, DAPI) in RAW 264.7 cells treated with DMXAA (10 µg/ml) for 0.5, 1, 1.5, 3, and 6 h. Scale bar: 10 µm. (**B**) Quantification of fluorescence intensity of endogenous STING1 within the Golgi (GOLGA2 area) using Columbus software (n = 4). (**C**) Quantification of STING1 (green) to GOLGA2 (red) fluorescence intensity ratio within the Golgi area using Columbus software (n = 4). (**D, E**) Representative immunoblot showing expression levels of the indicated proteins in RAW 264.7 cells incubated with DMXAA (10 and 50 µg/ml) for 1 and 2 h. (**F**) Representative immunofluorescence images of TFEB (green) in RAW 264.7 cells treated with DMXAA (10 µg/ml) for 0.5, 1, 1.5, 3, and 6 h. Scale bar: 50 µm (n = 3).

**Figure S3.** STING1-induced TFEB activation does not require TBK1 or IKBKE/IKKε. (**A, B**) Quantification of total protein levels from immunoblots in (Fig 3A) showing p-RPS6:total RPS6 (**A**) and p-EIF4EBP1:total EIF4EBP1 (**B**) ratios (n = 3). (**C**) Quantification of the number of LAMP1-positive puncta containing TFEB in iBMDMs treated with 10 µg/ml DMXAA, 250 nM torin-1 or torin-1+DMXAA for 1 h, as shown in (Fig 3C). Under the different conditions ≥130 cells were analyzed, and the following number of puncta were counted (Control: 1736, DMXAA: 1107, torin-1: 1419 and torin-1+DMXAA: 1897). Data are presented as mean ± SD. (ns) not significant and (****) p<0.0001 (One-way ANOVA followed by Dunnett’s multiple comparison post-test). (**D**) Representative immunoblot showing TFE3 and TFEB activation in wild-type (WT), *tbk1* KO, *ikbke* KO, or *tbk1* *ikbke* DKO iBMDMs treated with DMXAA (10 µg/ml) for 1 and 3 h (n = 3).

**Figure S4.** TFEB binds to the promoter of multiple immune genes in response to STING1 activation. (**A**) Gene Ontology (GO) analysis of Cellular Compartment for genes identified from ChIP-seq of endogenous TFEB in iBMDMs treated with DMXAA (10 µg/ml) for 2 h. (**B**) ChIP-Seq profile showing endogenous TFEB binding regions in the promoters of *Ccl4*, *Plau*, *Ccrl2*, *Zfp36*, *Ccl3*, *Tnfaip3*, *Cyba*, and *Nampt* in iBMDM cells treated with DMXAA (10 µg/ml) for 2 h. The transcription start site (TSS) is indicated. (**C**) Heatmap of differentially expressed TFEB immune genes in iBMDMs treated with DMXAA (10 µg/ml) for 3 h (n = 3).

**Figure S5.** STING1 signaling in TFEB- and TFE3-depleted iBMDM cells. (**A**) Representative immunoblot showing expression levels of TFE3, TFEB, p-STING1, STING1, p-TBK1, TBK1, p-IRF3, and IRF3 in iBMDM treated with DMXAA (5 µg/ml) for 4, 6, 8, and 12 h. All immunoblots are representative of three independent experiments (n = 3). (**B**) Quantification of total protein levels from immunoblots in (A) showing TFE3:GAPDH and TFEB:GAPDH ratios at 0 h as fold change (n = 3). (**C-E**) Quantification of total protein levels from immunoblots in (A) showing p-STING1:STING1 (**C**), p-TBK1:TBK1 (**D**), and p-IRF3:IRF3 (**E**) ratios after incubation with DMXAA for 4 h (n = 3). (**F**) Representative immunoblot showing expression levels of p-TBK1, TBK1, p-STING1, STING1, TFEB, TFE3, p-EIF4EBP1, and EIF4EBP1 in ARPE-19 cells treated with siRNAs duplexes targeted against *TFEB*, *TFE3*, *TFEB* and *TFE3* or not target (NT), and incubated with 10 µg/ml of STING1 Agonist-3 (diABZI)) for 1 h. (**G, H**) Quantification of total protein levels showing the ratios of p-STING1:STING1 and p-TBK1:TBK1 as fold changes from immunoblots in (F) (n = 2). Data in (B-E) are presented as mean ± SD. (ns) not significant, (*) p<0.05, (***) p<0.001, and (****) p<0.0001 (unpaired Student's t‐test). In (G-H), One-way ANOVA followed by Dunnett’s multiple comparison post-test.

**Figure S6.** TFEB and TFE3 regulate expression of autophagic and lysosomes genes in DMXAA-treated cells. (**A**) Principal component analysis (PCA) of genes with a q-value < 0.05 reveals distinct clustering of iBMDM control and *tfeb* *tfe3* DKO cells treated with DMXAA (10 µg/ml) for 3 h (n = 3). (**B**) Gene Ontology analysis of differentially expressed, downregulated genes in *tfeb* *tfe3* DKO cells, from RNA-Seq data analysis of iBMDM control and *tfeb* *tfe3* DKO cells treated with DMXAA (10 µg/ml) for 3 h, categorized by Cellular Compartment terms (n = 3). (**C, D**) Heatmaps of differentially expressed autophagy (**C**) and MTORC1 signaling (**D**) genes from RNA-Seq data of iBMDM control and *tfeb* *tfe3* DKO cells treated with DMXAA (10 µg/ml) for 3 h (n = 3). (**E**) Relative quantitative RT-PCR analysis of mRNA expression levels of *Lamp1*, *Atp6v1c1*, *Hexa*, and *Mcoln1* in control and TFEB-Flag expressing cells after treatment with DMXAA (10 µg/ml) for 3 and 6 h (n = 3). Data are presented as fold changes relative to 0 and 3 h (n = 3). (**F**) Quantification of total protein levels from immunoblots in Fig. 6C showing p-STING1:STING1 ratios in control and TFEB overexpressing iBMDMs treated with DMXAA (10 µg/ml) for 3 and 6 h (n = 3). (**G**) Quantification of protein levels showing IRF3:GAPDH ratios. Control and TFEB-Flag-expressing iBMDMs were treated simultaneously with DMXAA (10 µg/mL) and bafilomycin A_1_ (100 nM) for 3 h. Data in (E, F, and G) are presented as mean ± SD. (ns) not significant, (*) p<0.05, (***) p< 0.001, and (****) p<0.0001. (E and F) Two-way ANOVA and (G) One-way ANOVA.

**Figure S7.** TFEB expression modulates STING1-mediated immune response. (**A**) Relative quantitative RT-PCR analysis of mRNA expression levels of interferon-stimulated genes (ISGs) *Socs1*, *Ifit1*, and *Isg15* in control and TFEB overexpressing iBMDMs. Cells were treated with DMXAA (10 µg/ml) for 3 and 6 h (n = 3). (**B**) Representative immunoblot showing expression levels of the indicated proteins in in wild-type (WT) and *tbk1* *ikbke* DKO iBMDMs treated with DMXAA (10 µg/ml) for 1.5, 3 and 6 h (n = 3). (**C**) Quantification of total protein levels from immunoblots in (B) showing cleaved CASP3:GAPDH ratios in WT and *tbk1* *ikbke* DKO iBMDMs treated with DMXAA (10 µg/ml) for 3 h (n = 3). (**D**) Representative immunoblot showing expression levels of cleaved CASP3 in control and *tfeb* *tfe3* DKO iBMDMs treated with DMXAA (5 µg/ml) for 4, 6, 8, and 12 h (n = 3). (**E**) Quantification of total protein levels showing the ratio of cleaved CASP3:GAPDH at 8 h as fold increases from immunoblots in (D) (n = 3). (**F**) Quantification of LDH levels released into the extracellular media in *tfeb* *tfe3* DKO cells treated with DMXAA (10 µg/ml) for 6 h, relative to control iBMDMs (n = 3). (**G**) Quantification of total protein levels showing the ratio of cleaved CASP3:GAPDH in MEFs cells infected with HSV-1 (MOI 2.5) at 8, 16, 24, and 36 h as fold increases from immunoblots in Figure 7E (n = 3). Data in (A), (C), and (E-G) are presented as mean ± SD. (ns) not significant, (*) p<0.05, (**) p< 0.01 (***) p<0.001, and (****) p<0.0001. (A), (F), and (G) Two-way ANOVA. (C) and (E) unpaired Student's t‐test.
